# Evidences for a Role of Gut Microbiota in Pathogenesis and Management of Epilepsy

**DOI:** 10.3390/ijms22115576

**Published:** 2021-05-25

**Authors:** Jana Amlerova, Jan Šroubek, Francesco Angelucci, Jakub Hort

**Affiliations:** 1 Motol Epilepsy Center, Department of Neurology, 2nd Faculty of Medicine, Motol University Hospital, Charles University, 110 00 Prague, Czech Republic; 2 Department of Neurosurgery, Na Homolce Hospital, 150 00 Prague, Czech Republic; jan.sroubek@homolka.cz; 3 Department of Neurosurgery, Faculty of Medicine, Charles University, 500 03 Hradec Kralove, Czech Republic; 4 Memory Clinic, Department of Neurology, 2nd Faculty of Medicine, Motol University Hospital, Charles University, 110 00 Prague, Czech Republic; fangelucci@hotmail.com (F.A.); jakub.hort@gmail.com (J.H.); 5 International Clinical Research Center, St. Anne’s University Hospital Brno, 656 91 Brno, Czech Republic

**Keywords:** gut microbiota, epilepsy, treatment, ketogenic diet, physical activity

## Abstract

Epilepsy as a chronic neurological disorder is characterized by recurrent, unprovoked epileptic seizures. In about half of the people who suffer from epilepsy, the root cause of the disorder is unknown. In the other cases, different factors can cause the onset of epilepsy. In recent years, the role of gut microbiota has been recognized in many neurological disorders, including epilepsy. These data are based on studies of the gut microbiota–brain axis, a relationship starting by a dysbiosis followed by an alteration of brain functions. Interestingly, epileptic patients may show signs of dysbiosis, therefore the normalization of the gut microbiota may lead to improvement of epilepsy and to greater efficacy of anticonvulsant drugs. In this descriptive review, we analyze the evidences for the role of gut microbiota in epilepsy and hypothesize a mechanism of action of these microorganisms in the pathogenesis and treatment of the disease. Human studies revealed an increased prevalence of Firmicutes in patients with refractory epilepsy. Exposure to various compounds can change microbiota composition, decreasing or exacerbating epileptic seizures. These include antibiotics, epileptic drugs, probiotics and ketogenic diet. Finally, we hypothesize that physical activity may play a role in epilepsy through the modulation of the gut microbiota.

## 1. Introduction

### 1.1. Epilepsy

Epilepsy is a chronic brain disorder that affects millions of people around the world. It is characterized by the recurrent epileptic seizures, usually of short duration (seconds or a few minutes), which can manifest with or without the alteration of consciousness and/or with involuntary movements that affect only one part of the body [1]. When epilepsy is not associated with brain damage (i.e., lesions) it is called primary epilepsy, while when it is associated with brain lesions it is named secondary or symptomatic epilepsy [2]. Among the causes of symptomatic epilepsy, we can include head trauma, brain diseases, infectious diseases, perinatal damage, developmental disorders and metabolic disorders [3]. In any case, epilepsy must be treated with drug or surgical therapy [4]. Both therapies are not free from problems, such as the lack of response or the presence of side effects, even of serious entity in the case of antiepileptic drugs [5]. For these reasons, the search for the causes of epilepsy and its treatment still represents a major challenge for the scientific community. In recent years, there is much discussion on the role of intestinal microbes in the pathogenesis of many diseases of the central nervous system (CNS) [6], including epilepsy [7,8,9].

### 1.2. Gut Microbiota

The so-called intestinal (or gut) microbiota is a group of bacteria present in our intestine, and is a subset of the more general microbiota, but certainly the richest and most important [10]. It weighs about one and half kilograms and it is constituted by approximately 500 different species of bacteria, divided into 45 genera and 14 families.

The four dominant bacterial phyla in the human gut are *Firmicutes* (including *Lactobacillus*), *Bacteroidetes,* *Actinobacteria* and *Proteobacteria* (including *Escherichia*) [11]. Species from the genus *Bacteroides* alone constitute about 30% of all gut bacteria, suggesting that this genus is especially important in the functioning of the host [11].

Some species are very useful, such as *Bacteroides thetaiotaomicron* [12], which enormously increases the body’s ability to metabolize carbohydrates, while others can become harmful, such as *Clostridium difficile*, whose action is generally limited by the presence of other microbes, but which in some cases can cause diarrhea and fever [13].

The population of “good” gut microbes (which are the vast majority) also protects the host (i.e., humans) by producing a mucus that acts as a barrier between microorganisms and the cells forming the intestinal wall [14]. It also stimulates the inflammatory response and the immune defenses against external pathogenic agents [15]. For these reasons, the microbiome has become a field of extreme interest for all medical fields, because, unlike some factors that cannot be modified and that affect the onset of diseases (such as age and genetics) it is possible, at least in theory, to modify the microbiome composition. In neurological disorders, scientists consider the so-called gut–brain axis as a system capable of positively or negatively influencing brain functions through the activity of the microbiota [16,17].

## 2. The Gut–Brain Axis and Neurological Disorders

For a long time, scientists have recognized the fundamental role of communication between the gut and the brain in safeguarding human health. However, in the last two decades the idea has emerged that the trillions of microorganisms present in the gut are one of the key regulators of the gut-brain axis [10,17,18].

Hundreds of studies have examined the way by which intestinal microorganisms communicate with the brain and have identified a correlation between various neurological disorders [19], both age-related and neurodegenerative [20,21], and the microbiota.

Many studies indicate that interactions between the microbiota and the host in the intestine lead to the release of immune system molecules, neurotransmitters and microbial metabolites that can influence neuronal messages and possibly regulate brain functions and behavior. For example, it has been shown that the intestinal microbiota is able to modulate the enteric nervous system, a network of neurons that governs the functions of the gastrointestinal tract [18]. Furthermore, the microbiota has been shown to synthesize and respond to numerous neurotransmitters, including serotonin and GABA, which are involved in human behavior and cognitive activities [22].

**Figure 1:**
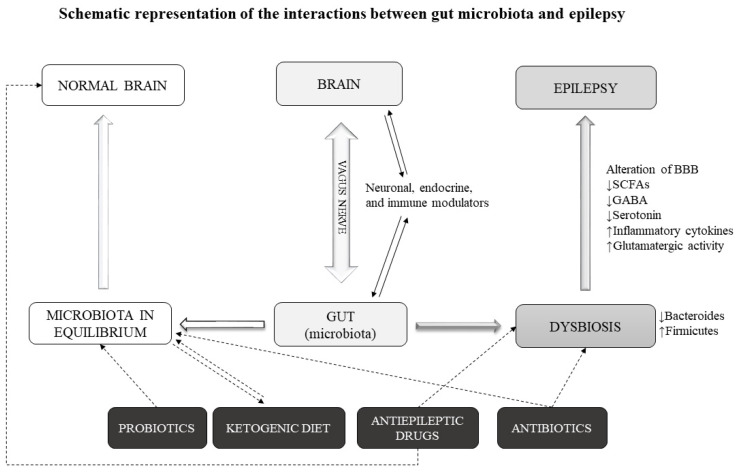
Schematic representation of the interactions between gut microbiota and epilepsy. Intestinal microorganisms can communicate with the brain in various ways, including the production of microbial metabolites, activation of the vagus nerve and modulation of the endocrine and immune response. In conditions of dysbiosis, alterations can occur that favor the onset of epileptic seizures. A reduction in short-chain fatty acids (SCFAs) alters the blood brain barrier, allowing the entry of toxins and inflammatory cytokines. Furthermore, a reduced production of neurotransmitters such as GABA and serotonin in the gut–brain axis can contribute to increase glutamatergic activity in the brain and favor the onset of seizure. External agents (such as antibiotics, probiotics, antiepileptic drugs and ketogenic diet) can modify the gut microbiota in a favorable or opposite direction to epileptic seizures.

Finally, many microbial byproducts such as secondary bile acids and short-chain fatty acids appear to be implicated in gastrointestinal functions, blood pressure regulation, circadian rhythm and neuroimmune function [23]. For example, in the case of disorders that affect the brain and behavior, such as Parkinson’s disease, reduced levels of short-chain fatty acids have been observed in the stool [24]. Communication between gut and brain may occur in various ways, including the production of microbial metabolites, activation of the vagus nerve and modulation of the endocrine and immune response (Figure 1).

Whether and how the microbiota can have long-term effects on brain functions is poorly understood. Some studies have shown that germ-free mice have altered levels of brain proteins and receptors that are thought to play an important role in neuroplasticity [25], and on anxiety and depression in humans [26]. Accordingly, the administration of probiotics has been shown to improve mood and anxiety in humans [27].

In addition, an altered microbiota profile has also been associated with neurodegenerative disorders, such as Parkinson’s and Alzheimer’s disease [21,28,29]. Fecal transplantation from Parkinson’s patients into germ-free mice caused motor deficits and neuroinflammation, two hallmarks of Parkinson’s [30]. Reduced levels of Firmicutes and Bifidobacterium, and a greater abundance of Bacteroidetes were observed in the intestines of patients with Alzheimer’s compared to healthy people [31]. Similarly, in the feces of patients with multiple sclerosis, the researchers found a high abundance of *Akkermansia muciniphila* and *Acinetobacter calcoaceticus* [32].

These data indicate the involvement of the gut microbiota in various neurological disorders. However, it is still unclear whether changes in the microbiota represent a key event for the development of these disorders.

## 3. Alterations of Gut–Microbiota in Epilepsy

We described how the microbiota can influence brain activity through various mechanisms and be involved in many neuronal pathologies. In this section, we will examine the changes of the gut microbiota in epilepsy.

### 3.1. Preclinical Studies

In a series of studies on rodent models of epilepsy, it has been shown how the microbiota can influence epileptic seizures [7]. In a study on rats, it was observed that under stressful conditions these animals are more susceptible to developing epileptic seizures [33]. Stress is able to alter the gut microbiota [34] and when the fecal contents of these rats were transplanted into non-stressed animals, the latter developed a greater sensitivity to epilepsy than controls [33].

In another recent study in mice, intestinal inflammation was found to increase pharmacologically induced convulsing activity [35]. Accordingly, the administration of anti-inflammatories reduces susceptibility to seizure and restores the effectiveness of antiepileptic agents.

In another study, it was observed that an intestinal infection induced by Gram negative bacteria (such as bacterium *B. fragilisor* lipopolysaccharide) can lead to the formation of cerebral cavernous malformations (CCMs), structural abnormalities in brain capillaries that predispose to stroke and seizures [36], in genetically susceptible mice. Furthermore, when these mice are raised as germ-free, they fail to form CCM lesions. These studies suggest that alterations in the microbiota gut can lead to an increase in seizure vulnerability, although this mechanism (or relationship) is not yet well understood.

### 3.2. Human Studies

In accordance with animal studies, a relationship between gut microbiota and epilepsy has been observed in humans. First, epileptic patients may present with altered microbiota [37]. Furthermore, it appears that patients with greater alterations of the microbiota composition are those with refractory epilepsy compared to those with drug-sensitive epilepsy and healthy controls [9,38]. In addition, differences in fecal composition between refractory epilepsy and healthy controls were also found in infant patients aged 1–4 years [39]. In another study in children aged 2–17 years, a fecal microbial α-diversity was observed among patients with refractory epilepsy and the healthy control parents [40]. In general, taxonomic analysis indicated that microbiota from children with refractory epilepsy displayed decreased relative abundances of Bacteroidetes and Proteobacteria and increased relative abundances of Firmicutes and Actinobacteria, when compared to control parent samples [41].

Overall, these human studies report alterations in the fecal microbiota of individuals with refractory epilepsy relative to varied non-epileptic controls [42]. Although these studies show limitations given by the differences among the groups of epileptic patients included and by the methods of investigation used, there seems to be an association between the composition of the gut microbiota and the susceptibility to epileptic activity (Table 1).

## 4. Possible Mechanism of Action of Gut Microbiota in Epilepsy

To fully understand the role of the gut microbiota, we need to analyze the phenomena that occur during an epileptic seizure. Nonetheless, it is difficult to establish a certain role for the gut microbiota in epilepsy, since the causes of the disease are still unknown (primary epilepsy) or as many as in secondary epilepsy.

During an epileptic seizure, the resistance of excitatory neurons to stimuli appears decreased for a certain period. This can occur due to changes in ion channels or the improper functioning of inhibitory neurons. This then results in a specific area from which seizures can develop [43]. A further mechanism leading to epilepsy may be due to the “up” regulation of excitatory neuronal circuits or the “down” regulation of inhibitory circuits, following brain damage [44]. Such secondary epilepsies occur through processes known as “epileptogenesis” [45]. “Epileptogenic threshold” is the term used to indicate the amount of stimulus needed for an attack to occur. In epileptic patients, this threshold appears much lower than in the healthy population. Impaired blood–brain barrier (BBB) may also be a causal mechanism, as it would allow substances in the blood to enter the brain [46].

The present data indicate that the gut microbiota can favor or reduce the epileptic manifestations. It is possible that this happens through the gut-brain axis. The presence of alterations in favor of the so-called bad bacteria could promote epilepsy through various mechanisms. Gut bacteria are responsible for the production of various substances that can alter the excitatory-inhibitory balance. These include cytokines, and metabolites serving as neuromodulators, such as short-chain fatty acids (SCFAs), γ-aminobutyric acid (GABA) and serotonin precursors [47,48]. We know that seizures are due to an imbalance of the excitatory–inhibitory balance. When the neurotransmitter GABA is reduced, the threshold for the onset of a seizure is lowered [44,49].

A condition of dysbiosis can lead to an influx of toxins and cytokines through an alteration of the BBB. Under these conditions, there is a reduced production of SCFAs and GABA. SCFAs act as anti-inflammatories and their reduction causes an alteration of the BBB [35]. Furthermore, a reduced influx of GABA in the gut–brain axis can contribute to the onset of seizure. In addition, even a reduced intake of serotonin can cause epileptic manifestations, as shown in many studies [50]. Therefore, we can hypothesize a mechanism based on the alteration of the gut microbiota and a consequent alteration of neurotransmitters, including GABA, serotonin and glutamate in a direction that can favor epileptic seizures. We cannot affirm that dysbiosis is the cause (or one of the causes) of epilepsy but we can say that it can contribute to the onset of epileptic seizures through these mechanisms (Figure 1).

According to this hypothesis, the presence of good bacteria in the gut microbiota has a therapeutic effect on epileptic phenomena. In fact, in these conditions a normal production of neurotransmitter metabolites such as GABA can contribute to increasing the convulsion threshold.

Nonetheless, since the gut microbiota composition is so complex, it is difficult to pinpoint certain bacteria as the most beneficial in epilepsy.

### Preclinical and Human Studies

Some studies suggest that in refractory epilepsy there is an increase in Firmicutes and a decrease in *Bacteroides* relative to controls. These alterations have been reported either in adult or infants with refractory epilepsy [38,39,51,52]. *Lactobacillus* population is also suspected to have a beneficial role in epilepsy. *Lactobacillus* can influence brain function through the modulation of GABA, as shown in rodent models [53]. Moreover, it has been demonstrated in animal models of epilepsy and in human epileptic patients that probiotic treatment aimed at restoring gut microbiota equilibrium have beneficial effects on epileptic symptoms by increasing GABA in animals [54] and the levels of *Bifidobacteria* and *Lactobacillus* in humans [38,55].

We therefore can come to a conclusion on the role of microbiota in epilepsy. Alterations of the microbiome in one sense or another can suppress or promote seizures. For these reasons, we are witnessing phenomena in which agents that modify the gut microbiota, such as antibiotics, can both favor epileptic seizures [56] or lead to their reduction [57,58]. However, it is not clear whether the alterations in the microbiota in epileptic patients are congenital or possibly induced by other external factors, such as drug treatments or antibiotics.

## 5. External Agents Modifying Microbiota in Epilepsy

If the gut microbiota plays a role in epilepsy, its modulation could have effects on the disease course. Gut microbiota alterations in humans can occur for a number of reasons: dietary, physical and physiological factors. In epilepsy, however, we can identify external agents that are able to modify the gut microbiota. The data supporting this notion are already quite numerous and concern the use of medicines or treatments that act on these intestinal bacteria. Among these treatments, we can include the aforementioned antiepileptic drugs and antibiotics. Other treatments that can modify epilepsy course possibly through an action on gut microbiota include the ketogenic diet and probiotics (Figure 1). In this section, we review the data on these microbiota-modifying agents in epilepsy.

### 5.1. Antibiotics

It is well known that antibiotics are able to alter the gut microbiota [59]. Antibiotics represent the most direct and effective way of targeting intestinal microbes.

#### 5.1.1. Preclinical Studies

Evidence gathered from in vitro and in vivo studies suggests that a course of short-term antibiotics can substantially change the gut microbiota composition [60,61]. In addition, there are also documented alterations in the level of brain activity. Antibiotics have been associated with alterations in neurogenesis, apoptosis and synaptic pruning [62]. It is possible that the alterations in microbiota and brain function caused by antibiotics are related. In fact, treatments with probiotics and prebiotics are able to restore cognitive and behavioral deficits in rodents and humans [63,64]. A metabolomics study in rats exposed to penicillin demonstrated not only changes in the gut microbiome composition, but also a decrease in vitamins, bile acids and conjugated urinary metabolites, suggesting that absorption of drugs may be affected by variation in the gut microbiome [65].

#### 5.1.2. Human Studies

With regard to epilepsy, the use of antibiotics can cause epileptic seizures [66]. Numerous studies indicate that some antibiotics, such as unsubstituted penicillins, fourth-generation cephalosporins, imipenem and ciprofloxacin, increase the risk of symptomatic seizures [7,66], although this risk has recently been quantified as low to very low (evidence Class III-IV) [56]. In any case, close monitoring of dosage and EEG activity is still recommended in subjects predisposed to epileptic seizures. These antibiotics might also lower the levels of some antiepileptic medications [67] and might have direct cortical effects or alter the microbiota.

Thus, antibiotics trough modification of microbiota composition are potentially implicated in the etiology and treatment of seizures [56,57]. However, as we have already said, the use of antibiotics can have positive effects on epilepsy. This hypothesis is based on some data, of which the most significant is a recent study from 2018, where six patients with drug-resistant epilepsy attained temporary seizure freedom after different antibiotic treatment [57]. In addition, when the antibiotics were stopped, the seizures started again. The authors hypothesized a role of gut bacteria in explaining the beneficial effect of antibiotics on seizure, without however providing direct evidence for this theory.

### 5.2. Antiepileptic Medications

There is some data indicating that some anticonvulsant agents can alter the composition of the gut microbiota. Antiepileptic drugs can have antimicrobial activities and can therefore interact with the gut microbiota. However, these data are currently derived from in vitro studies or in animal models and the results, positive or negative, on the composition of the gut microbiota are unclear.

#### Preclinical Studies

In the model organism soil collembolan (*Folsomia candida*) it was shown that the antiepileptic drug carbamazepine alter the gut microbiota composition [68]. In vitro, lamotrigine was shown to inhibit *E. coli* growth [69]. In a study conducted on rats with various psychotropic substances, lithium and valproic acid (VPA) were found to alter the composition of the gut microbiota [70]. In details, at the phylum level, lithium induced a significant increase in Actinobacteria and a decrease in Bacteroidetes; VPA induced an increase in Actinobacteria, Firmicutes and a decrease in Bacteroidetes. In addition, VPA may affect SCFAs: VPA administration induced a significant decrease in the levels of propionate and butyrate while augmenting the levels of isovalerate. Moreover, VPA used as a model of autism in rats was able to reduce fecal microbial richness, change the gut microbial composition, and alter the metabolite potential of the fecal microbial community in a pattern similar to that seen in patients with autism [71]. Other animal studies have revealed microbiota changes in the offspring of mothers treated with VPA; for example, decreased fecal Firmicutes and increased Bacteroidetes in pups of treated mothers [7,72].

On the other hand, the gut microbiome can affect drug metabolism altering absorption, bioavailability and efficacy of the medication, through various direct and indirect mechanisms [73,74]. Drugs are transformed to bioactive, inactive, or toxic metabolites by microbial direct action or host-microbial cometabolism. These metabolites might be responsible for therapeutic effects or side effects induced by these drugs [75]. Animal studies have shown interactions between some of these drugs and microbiota [7,8]. Zonisamide and clonazepam are metabolized by microbiota, and antibiotics can alter levels of their metabolites [73,76]. Furthermore, it seems that an intestinal infection in epileptic mouse models can influence the activity of antiepileptic drugs such as VPA, probably through an action on the gut microbiota [35]. Thus, alteration in the microbiome composition may affect absorption and metabolism of drugs, thereby influencing their efficacy. How these microbial changes relate to drug efficacy is not clear. Moreover, it is also not clarified whether these drugs affect directly the gut microbiota (i.e., they reach the caecum) or indirectly (i.e., through gut–brain signaling). The impact of antiepileptic drugs on the microbiota and, vice versa, the impact of microbiota on drug metabolism and function require further investigation.

### 5.3. Ketogenic Diet

The ketogenic diet is a diet that drastically reduces carbohydrates, while increasing proteins and especially fats. The main purpose of this imbalance in the proportions of macronutrients in the diet is to force the body to use fat as an energy source.

Numerous studies associate this diet with an improvement in epilepsy [77]. Since the last century, it has been observed that fasting has a “sedative” effect on epileptic seizures. Over time, the idea of resorting to the ketogenic diet as a therapeutic option for these patients was born. At the basis of the mechanism of action of the dietary pattern in epilepsy, there seem to be the ketone bodies produced to make up for the lack of glucose [77]. However, the actual mechanism of action is still partially unknown. This diet induces a metabolic condition known as physiological ketosis. The ketone bodies synthesized by the liver–acetone, acetoacetate and D-beta-hydroxybutyrate are used to “feed” the brain. When sugars become too low, cells draw energy from fat. All, except neurons, which need ketone bodies.

In recent years, many studies have highlighted the effects of the ketogenic diet on the gut microbiota and linked the latter to the antiepileptic effects of this diet [9,78]. It has been observed that the diet alters the composition of the microbiota [79], but the question was raised as to whether the effects of the diet on epilepsy are directly due to changes in the microbiota towards an antiseizure effect [80,81].

#### 5.3.1. Preclinical Studies

It has been demonstrated, for example, that in mice treated with antibiotics or germ free, the ketogenic diet has no effect on seizures but the administration of probiotics restores the effects of the diet by regulating the glutamate-GABA axis [80]. It was also shown in epileptic mice that the administration of *Lactobacillus fermentum* MSK 408 has the same antiepileptic effects as the diet, suggesting that direct modulation of the gut microbiota could be a more convincing choice [82].

#### 5.3.2. Human studies

In a human study, it was found that the effects of the ketogenic diet in infants with refractory epilepsy are associated with the normalization of the gut microbiota [39].

Furthermore, scientists are trying to understand if the effects of the diet can be increased by administering, for example, probiotics, in patients in whom the diet has no effect. Indeed, in more recent studies, it was shown that the ketogenic diet might have negative effects on the composition of gut microbiota, namely a reduction in *Bifidobacteria* and an increase in *Escherichia coli* [40,79]. Despite this, the present data indicate that the association between gut microbiota and ketogenic diet exists. This result is once again in favor of a predominant role of the gut microbiota in epilepsy [77,78,81].

### 5.4. Probiotics

Probiotics can also alter the gut microbiota and stabilize microbial communities. Normalization of gut microbiota composition may have beneficial effects on epileptic seizures. Probiotics have been found to have beneficial effects on epileptic symptoms by increasing GABA in animals [54] and the levels of *Bifidobacteria* and *Lactobacillus* in humans [38,55].

#### Human Studies

In this regard, some studies in humans have reported encouraging results. One study showed that administration of probiotics (*Saccharomyces boulardii* or *Lactobacillus casei*) on neonates affected by rotavirus was associated with a 10-fold decreased risk for seizures as compared to non-treated infected controls [83]. The authors proposed that *S. boulardii* reduces seizures through inhibition of rotavirus structural protein 4, a viral enterotoxin that increases reactive oxygen species and white matter injury, or through suppressing the inflammatory response overall. Another study on drug-resistant symptomatic epileptic patients showed that a probiotic cocktail administration was associated with a >50% reduction in seizure frequency in 29% of patients and 77% of them maintained reduced seizure frequency 4 months after discontinuation [55]. Whether therapeutic effects of probiotics are mediated by modifications in gut microbiome is not yet clear. However, there is an interesting study that demonstrated how fecal microbiota transplantation was able to eliminate seizure in a patient affected by epilepsy and concomitant Crohn’s disease, even after the withdrawal of antiepileptic drugs [84].

Overall, these studies suggest that probiotics might constitute a potential supplementary therapy in patients with drug-resistant epilepsy.

### 5.5. Physical Activity

An interesting addition to this topic concerns the role of physical activity in epilepsy. It is currently unclear whether physical activity can improve or worsen the disease condition.

#### 5.5.1. Preclinical Studies

Preclinical studies have indicated several mechanisms of physical activity to mediate the inhibitory/excitatory balance to reduce seizure susceptibility, including modulation of neurotransmitters, neurotrophins and brain metabolism. However, some studies point to another possible mechanism. In experiments of fecal transplantation in mice, it has been observed that colonization of mice with a gut microbiota from exercise-trained mice led to modification of gut microbiota in the host that make the mice more resistant to chemically-induced colitis infections [85]. In another study, it is shown how sporting activity is able to alter the gut microbiota in a mouse model of colitis [86]. Interestingly, it is shown how spontaneous activity protects while forced activity increases colitis infection [86]. In humans it has been seen that athletes have a higher diversity of gut microorganisms as compared to non-athletic controls [87]. It is suggested that exercise increases gut motility, which may increase the shedding of loosely bound microbes in the gut epithelium. This effect promotes the growth of other commensals that participate in the development of healthy mucosal immunity and provide benefits in gut tissue and beyond [88].

#### 5.5.2. Human Studies

Some studies report that physical activity can exacerbate epileptic seizures through a number of factors including hyperthermia, hypoglycemia, hyponatremia, hypoxia, hyperventilation, fatigue and stress of competition. However, in some other studies, physical activity was shown to have beneficial effects [89,90]. For instance, a decreased number of seizures were reported in women with intractable epilepsy submitted to 15 weeks of aerobic physical training [91]. Two studies reported no increase in seizure frequency after 4 weeks [92] or 12 weeks of a physical exercise program [93].

Several hypotheses have been suggested to explain these positive effects on epilepsy. Intensive exercise increases blood lactate, which produces metabolic acidosis. Therefore, reduced epileptogenic EEG activity following intensive physical effort may be related to an increase in GABA concentration due to metabolic acidosis [94]. This view is supported by the fact that acidosis increases GABA concentration, which, in turn, reduces neuronal excitability and may prevent seizures; conversely alkalosis may precipitate seizures by the opposite mechanism [95].

**Figure 2:**
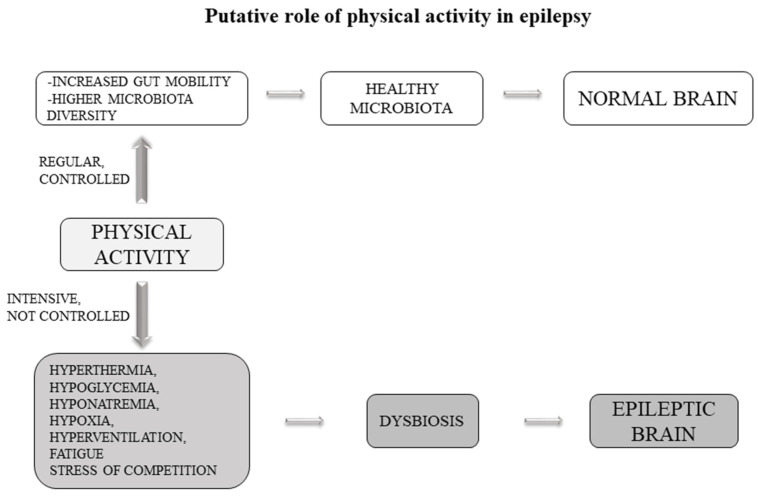
Putative role of physical activity in epilepsy. Physical activity can induce or reduce epileptic seizures. In case of intense (or inadequate) physical activity in predisposed subjects, some side effects (such as stress, hyperventilation, fatigue, hypoglycemia, etc.) can alter the gut microbiota towards a condition of dysbiosis and favor epileptic seizures. Adequate and regular physical activity can instead promote gut mobility and create higher microbiota diversity, leading to a reduction or inhibition of seizures.

Based on the current knowledge, we can hypothesize that the effects of physical activity on epilepsy may also be due to the gut microbiota (Figure 2). This hypothesis is fascinating and opens the way for further study towards the direction of an adjuvant therapy based on physical activity. Another example, in theory, of how it is possible to try to reduce the administration of antiepileptic drugs towards the use of more physiological therapies.

## 6. Future Directions

The treatment of epilepsy offers a range spectrum of options. Despite this, almost 25% of patients show signs of drug resistance [96] and antiepileptic drugs can have serious side effects [97]. In such a context, if modulation of the gut microbiota could help to ameliorate the treatment outcome, it would be of great help. In this review, we highlighted the data on the role of gut microbiota in epilepsy and several methods for modulating the gut microbiota. The aforementioned antibiotics [57], probiotics [42,53,55] and the ketogenic diet [81].

There are basically two roads to explore: (1) whether patients resistant to antiepileptic drugs can benefit from additional treatment that acts on the gut microbiota [42,55,98]; and (2) explore the possibility of using microbiota gut modulators as a primary treatment for epilepsy [99], thus eliminating antiepileptic drugs and/or the ketogenic diet, with the benefits that this may entail [100].

Both roads need further studies to better define the role of the gut microbiota in epilepsy. In particular, it is necessary to establish which alterations are present in epileptic patients in general and in those refractory to drugs in particular. Once the alterations in the composition or function of gut microbiota in epilepsy are clarified, this information could be used to treat patients with more targeted interventions (e.g., ketogenic diet, probiotics, prebiotics and physical activity).

## 7. Conclusions

In this review, we summarized the experimental evidence for the role of the gut microbiota in epilepsy. From the information from both animal models and human studies, it is well established that gut microbiota changes are present in epilepsy. Directions of changes are difficult to be ascertained, although many studies point to an increased prevalence of Firmicutes relative to *Bacteroides* in patients with refractory epilepsy.

Exposure to various compounds can also change the intestinal flora in a positive or negative sense, decreasing or exacerbating epileptic seizures. These include antibiotics, epileptic drugs, probiotics and the ketogenic diet. These modifying agents can be manipulated to obtain an improvement of epileptic symptoms through their action on gut microbiota. Finally, we hypothesized that physical activity may play a role in epilepsy through the modulation of the gut microbiota.

It is at present not clear whether microbiota changes are a consequence of epileptic state or can be the cause (or one of the causes) of the disease. In the second hypothesis, a direct intervention toward normalization of gut microbiota composition may represent a new possible therapeutic approach.

## Figures and Tables

**Table 1:** Alterations in gut microbiota composition in subjects affected by epilepsy treated with antiepileptic drugs and/or ketogenic diet.

Study Participants	Type of Epilepsy and Treatment	Alteration in Gut Microbiota	References
Adults with drug resistant or drug sensitive epilepsy	Epilepsy treated with antiepileptics	Variations in the fecal microbiota composition between patients with different clinical prognoses and between patients and controls	[37]
Adults with drug resistant or drug sensitive epilepsy	Epilepsy treated with antiepileptics	Increased abundance of rare flora in the fecal composition of drug resistant epileptic patients vs. drug sensitive patients and controls. Reduction of Bifidobacteria and Lactobacillus in drug resistant patients	[38]
Infants aged 1–4 years with refractory epilepsy	Refractory Epilepsy treated with ketogenic diet	Higher gut microbiota diversity in healthy infants. Increased Proteobacteria and Firmicutes in refractory epilepsy	[39]
Children aged 2–17 years with refractory epilepsy	Refractory Epilepsy treated with ketogenic diet	Fecal microbial α-diversity in patients with refractory epilepsy and the healthy control parents	[40]
Children aged 2–11 years with refractory epilepsy	Refractory Epilepsy treated with ketogenic diet	Lower alpha diversity, decreased levels of Firmicutes and increased levels of Bacteroidetes after ketogenic diet therapy.	[41]
